# Consequences of Repeated Blood-Brain Barrier Disruption in Football Players

**DOI:** 10.1371/journal.pone.0056805

**Published:** 2013-03-06

**Authors:** Nicola Marchi, Jeffrey J. Bazarian, Vikram Puvenna, Mattia Janigro, Chaitali Ghosh, Jianhui Zhong, Tong Zhu, Eric Blackman, Desiree Stewart, Jasmina Ellis, Robert Butler, Damir Janigro

**Affiliations:** 1 Cerebrovascular Research, Cleveland Clinic Lerner College of Medicine, Cleveland, Ohio, United States of America; 2 Departments of Neurosurgery and Cell and Molecular Medicine, Cleveland Clinic Lerner College of Medicine, Cleveland, Ohio, United States of America; 3 Molecular Medicine – Cleveland Clinic Lerner College of Medicine, Cleveland, Ohio, United States of America; 4 Flocel, Inc. Cleveland, Ohio, United States of America; 5 Baldwin-Wallace College, Berea, Ohio, United States of America; 6 Department of Quantitative Health Sciences, Cleveland Clinic, Cleveland, Ohio, United States of America; 7 University of Rochester Medical Center, Rochester, New York, United States of America; Georgia Health Sciences University, United States of America

## Abstract

The acknowledgement of risks for traumatic brain injury in American football players has prompted studies for sideline concussion diagnosis and testing for neurological deficits. While concussions are recognized etiological factors for a spectrum of neurological sequelae, the consequences of sub-concussive events are unclear. We tested the hypothesis that blood-brain barrier disruption (BBBD) and the accompanying surge of the astrocytic protein S100B in blood may cause an immune response associated with production of auto-antibodies. We also wished to determine whether these events result in disrupted white matter on diffusion tensor imaging (DT) scans. Players from three college football teams were enrolled (total of 67 volunteers). None of the players experienced a concussion. Blood samples were collected before and after games (n = 57); the number of head hits in all players was monitored by movie review and post-game interviews. S100B serum levels and auto-antibodies against S100B were measured and correlated by direct and reverse immunoassays (n = 15 players; 5 games). A subset of players underwent DTI scans pre- and post-season and after a 6-month interval (n = 10). Cognitive and functional assessments were also performed. After a game, transient BBB damage measured by serum S100B was detected only in players experiencing the greatest number of sub-concussive head hits. Elevated levels of auto-antibodies against S100B were elevated only after repeated sub-concussive events characterized by BBBD. Serum levels of S100B auto-antibodies also predicted persistence of MRI-DTI abnormalities which in turn correlated with cognitive changes. Even in the absence of concussion, football players may experience repeated BBBD and serum surges of the potential auto-antigen S100B. The correlation of serum S100B, auto-antibodies and DTI changes support a link between repeated BBBD and future risk for cognitive changes.

## Introduction

A recent review of the current practice of concussion management and time until return to play in the National Football League over a 5 year period from 1996–2001 showed that 50% of players returned to the same game after sustaining a concussion. The number of sub-concussive head hits has been estimated to be of the orders of hundreds per season [1], [2]. Clinically, concussions can produce symptoms that are of short or long duration lasting from several minutes to several months. Severe post-concussive symptoms have been reported to last several years in some athletes and have caused numerous athletes to retire from sports altogether. Short-term outcomes include cognitive defects, but in some cases there is evidence of permanent or subtle neurologic decline even after just one injury.

While football-related concussions are becoming increasing recognized, the mechanisms (and therapies) remain poorly understood. Blood-brain barrier disruption (BBBD) or increased permeability of the brain vasculature has been linked to a variety of neurological disorders including seizures, Alzheimer's disease, stroke, and traumatic brain injury [3]. In general, BBB failure can be pathogenic acutely or after a delay lasting anywhere from hours to years [4]–[6]. BBBD after trauma causes both immediate and delayed cognitive sequelae [7], [8]. Consequences of BBBD may be even more deleterious when accompanied by an autoimmune response, as in multiple sclerosis [9]. Loss of BBB integrity supports central nervous system (CNS) antigens unmasking and triggers a peripheral immune response. Circulating autoantibodies against CNS antigen become pathogenic to the brain when the BBB allows their access into the brain [10].

A link between BBBD and neurological diseases has been difficult to establish because of the lack of reliable means to non-invasively measure cerebrovascular function and integrity. A longitudinal link and a cause-effect relationship between BBBD and neurological diseases were made possible by use of peripheral markers of BBB dysfunction, such as the appearance in serum of the astrocytic protein S100B [11], [12]. The sensitivity of S100B for BBB leakage is comparable to measurements of albumin in cerebrospinal fluid (CSF, [13], [14]) and S100B levels correlate with the presence or absence of enhancements in MRI scans [15], [16]. BBBD occurs immediately after traumatic brain injury (TBI) [13], [14], [17]. S100B serum levels have been used to study BBBD after TBI and this marker is used in emergency room settings to rule out mTBI [18], [19]. The biological consequences and, in general, the functions of S100B remain elusive.

The pathophysiological significance of S100B may extend beyond the timeframe of its appearance in serum after BBBD. For example, it has been shown that in Alzheimer's patients autoantibodies against S100B and other brain proteins can be measured [20]–[23]. We therefore tested the hypothesis that players who experience repeated serum S100B increases due to BBBD also develop autoimmunity against this CNS protein.

American football (professional or amateur) is one of the sports with the highest risk for head trauma and brain injury. Several studies have shown that football players are at varying risks for TBI depending on their field position [1], [2]. While concussions are routinely diagnosed, there is evidence that most sub-concussive episodes remain undiagnosed [18], [19], [24]–[27]. The short and long-term clinical implications of sub-concussive injuries are unknown. Since TBI is associated with BBBD, and given the fact that S100B in serum is a sensitive indicator of BBBD, we wished to test the hypothesis that serum S100B elevation can be measured in football players following sub-concussive head hits (SHH). However, since other “gold standards” exist to diagnose SHH and mTBI we wished to compare BBB damage data to findings obtained by MRI-DTI and cognitive assessment. We also wished to measure and compare the effect of an immune response against brain protein such as S100B in the context of sub-acute consequences of SHH.

## Materials and Methods

### Demographics and design

**Figure 1:**
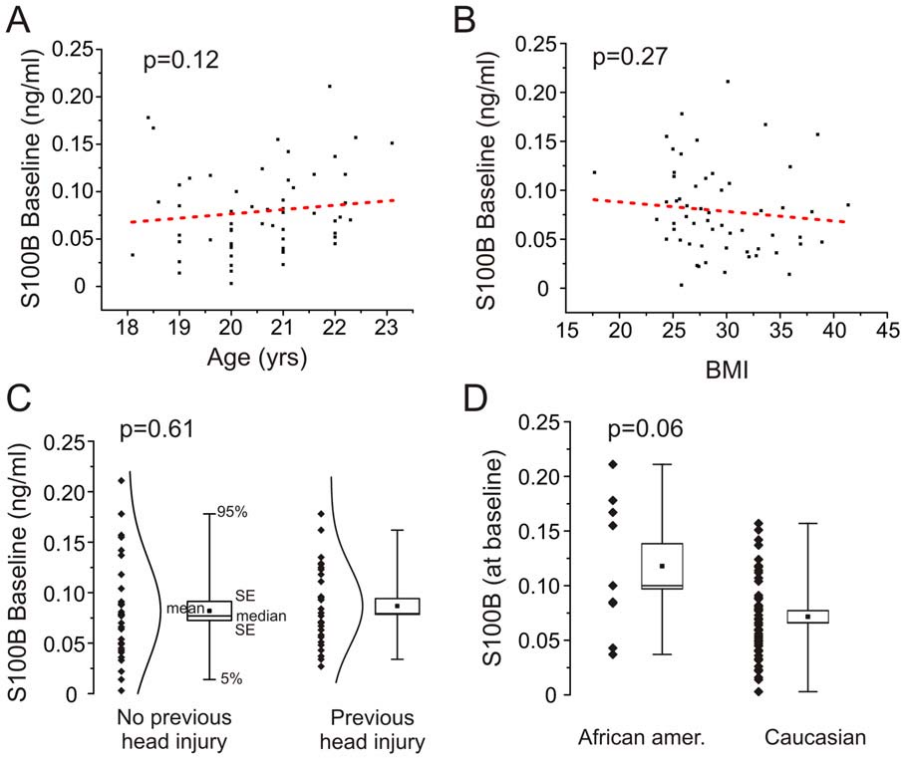
Demographics of the Cleveland players enrolled in the study. Please also see Table 1 and Table S1. S100B serum levels measured at baseline (prior to any games) where independent from age (**A**) and body mass index (**B**). History of previous concussion did not affect S100B baseline values (**C**). Baseline S100B levels were higher in African-American compared to other races (**D**).

**Figure 2:**
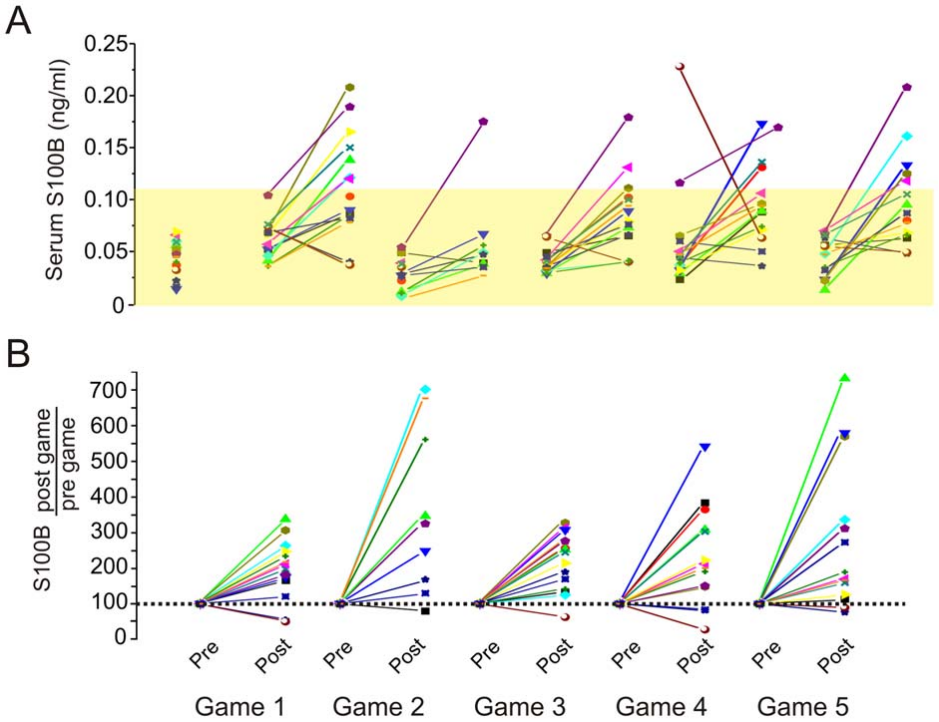
Pattern of S100B changes in players during a season. A ) and **B**) refer to absolute and normalized S100B serum levels respectively. S100B serum levels were measured at baseline (prior to any football-related activity), pre- (day before the game) and post-games (within one hour from the end of the game). The shaded region indicates the ceiling for blood-brain barrier disruption (0.12 ng/mL). The threshold for blood-brain barrier disruption derives from previous work, and clinical standard used in the EC for mTBI. The data are consistent across techniques, so a comparable threshold is seen when comparing BBBD measured by albumin coefficient against S100B [1], or when comparing gadolinium extravasation to S100B [2]; [3], or contrast-CT to S100B [4]. See text for details.

**Figure 3:**
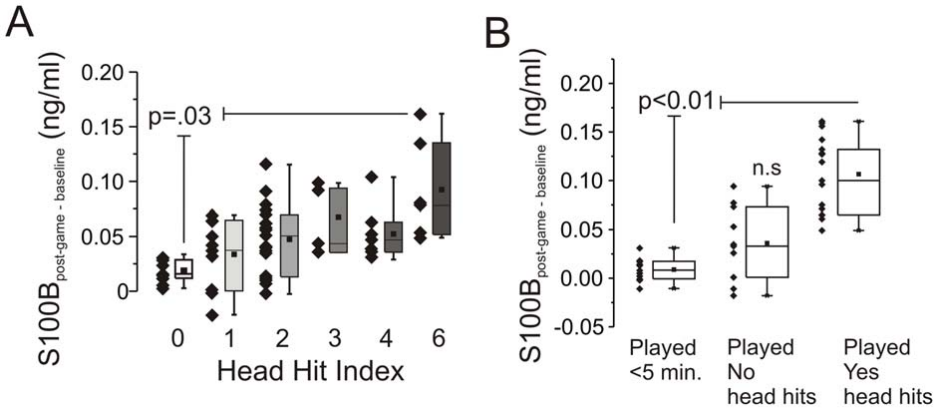
S100B increase in serum after games correlates with extent and number of head hits. **A**) S100_post-game – baseline_ levels are significantly correlated to HHI scores (see MethodsMethods and Table S2). A statistically significant difference was found between HHI 0 and 6. Moreover, when S100_post-game – baseline_ values were plotted against HHI (0 to 6 as a continuous variable) a statistically significant correlation was observed (p<0.05; R^2^ = 0.51). **B**) S100_post-game – baseline_ values are elevated only in players who experience head hits. Measurements were obtained by film game review.

All patients signed an informed consent according to institutional review protocols at The Cleveland Clinic Foundation and the Declaration of Helsinki. Human research was conducted *as per* Institutional Review Board (IRB) guidelines (approved protocol at Cleveland Clinic IRB 4406 – Dr. Janigro PI; University of Rochester IRB 37271 – PI Dr. Bazarian). Players were selected among those participating to the local football college tournaments (Cleveland, OH and Rochester, NY, USA). Prior to consent, introductory seminars were delivered to the colleges by DJ, NM and JB to explain the modality and the scope of the research. A written consent form was used to enroll players at the respective colleges. Players' demographic data (age, race, height, weight, history of previous concussion) were collected as per consent form and secured in a database available to the PIs (DJ/JB) only. Demographic categories were collected to rule out confounding factors (e.g., correlation between serum level of markers, race and body mass index; see Figure 1). We did not control for other confounding variables such as socio-economic status, nutrition or environmental exposures. S100B ELISA data were de-identified and secured in a data base available to the PI only (Figures 2 and S1). Blinded movie analysis (Figure 3) was performed by three operators unaware of the S100B serum data. Post-game interview for number and severity of head hits (Figure 3) was performed by JE and data delivered to DJ. We enrolled players from two Northeast Ohio Varsity College Teams and a college team at the University of Rochester, NY.

At the Cleveland site, we enrolled a total of 57 players. Thirty players gave blood samples 24 hours before the game, 1 hour after and 24 hours after the game. 27 players gave blood samples at baseline (prior to any football related activity), 24 hours before and 1 hour after 5 consecutive home games. Complete season data were available for 15 players; other players were absent for occasional blood draws and their data is incomplete. Game film review, Head Hit Index (HHI) analysis, pre- and post-game serum S100B and anti-S100BAb measurement were performed in these players.

At the University of Rochester site, 10 players were enrolled. In these players autoantibodies and S100B were measured at the beginning and end of season. Diffusion tensor imaging (DTI) measurements (see below) were also obtained in these players at the same time pre and post-season. The DTI auto-antibody experiments were performed as part of a separate IRB protocol that did not include blood sampling for S100B.

### Blood collection and S100B measurements

Blood samples were collected in red cap Vacutainer sterile tubes (BD Bioscience) and the serum was separated by centrifugation (2000 RPM, 10 minutes). Samples were de-identified and assigned an internal ID matching other testing. Serum samples were then stored at −80°C. Serum ID matched consent forms, player interview and game film review. S100B measurements were performed using an ELISA kit (98 wells, anti-human S100B, Diasorin) and reading done using a multi-plate fluorescent reader. Fluorescent signals were converted into ng/ml *as per* standard curve concentrations.

### Serum S100B auto-antibody measurement

Maxisorp ELISA 96 wells plates were coated with a PBS solution containing S100B protein (human brain, catalog number-559291, EMD Chemicals). Optimization of this ELISA was performed by testing two concentrations of S100B protein (1 or 5 µg per well). No significant differences were observed at these two concentrations of S100B coating. An S100B monoclonal antibody (catalog number: Q86610M, Meridian Life Science Inc.) was used as a calibration standard to allow the manipulation of AU values into either concentrations (µg/ml) or titer (number of dilutions). Standard curves were obtained with 100 µl of serially diluted S100B monoclonal antibodies (Figure S2). In a typical experiment, the relationship between absorbance and standards were then fitted by a polynomial equation: (y = 0.244−0.92X +0.89 X^2^ where y is expressed in µg/ml and x is absorbance. Table S3 and Figure S2 report these data in a graphical format.

Plates were coated overnight at 4C with S100B protein (1 µg/well). Wells were then washed 3 times with PBS. Subsequentially, 100 µL of a 1% BSA blocking solution was added in each well and incubated for 2 hours at room temperature. Wells were then washed 3 times with 200 µL of PBS containing 0.05% Tween 20. Serum samples and standards were added and incubated (1 hour at room temperature). Samples were then aspirated and wells washed 3 times using 200 µL of PBS containing 0.05% Tween-20. Subsequentially, 200 µL of HRP (horseradish-peroxidase) goat anti-mouse IgG and 200 µL of HRP goat anti-human were added to the standards and serum samples respectively. After 1 hour incubation at room temperature wells were washed 3 times with 200 µL of PBS containing 0.05% Tween-20. Finally, 100 µL of OPD solution was added and the reaction allowed for 30 minutes at room temperature. The reaction was stopped by adding 100 µL of 2.5 M Sulfuric acid. Samples were analyzed using an ELISA plate reader at 490 nm.

### Game film review

Games were filmed using 4 cameras; videos were analyzed to detect and count head or soft tissue contacts. Two blinded operators performed film review. Intra-observed and inter-observer variation in film analysis was taken into account by comparing the data obtained by the two operators. Based on film analysis, players were grouped into the following categories: a) did not play (or played <5 minutes); b) played but did not experience any significant head hits or c) played and suffered repetitive head hits.

### Head Hit Index (HHI)

A questionnaire was administered to each participant within 30 minutes following the completion of each game. HHI is not a tool to identify concussions but rather to quantify the occurrence of head hits. The questionnaire was design by two doctors specialized in sport medicine and authors of this manuscript (see Table S2). This questionnaire intended to be feasible enough to administer to larger numbers of participants given the time and space limitations of a collegiate locker-room setting. The questionnaire included parameters previously recognized in the literature as possible important predictors in head injury and was developed with the aid of the authors and concussion experts. Players enrolled in this study were asked to track the frequency and severity of significant collisions they experienced during each game. The questionnaire consisted of: 1) number of collisions experienced; 2) number of episodes of contact involving the head; 3) number of significant episodes of contact to the head; and 4) presence of acute symptoms (e.g., headache, neck pain, nausea). In the players enrolled in this study, no obvious loss of consciousness/awareness or acute traumatic episodes of dizziness were detected by the supervising team physicians during the events of interest. A Head Hit Index (HHI), ranging from 0 to 6, was derived from (a×b) where a) is the number of head collisions and b) is the overall severity of these head hits (see Table S2). Two players were excluded from the analysis in Figure 3 because of concussion related symptoms reported during pre-season games and because of persistent inconsistencies between film review results (several head hits observed by the two operators), self-reporting assessment (players did not report any experience of head hits) and blood S100B post-game elevations. For the rest of the players film review results correlate with HHI scoring (*not shown*).

### Computerized cognitive testing

Cognitive testing was performed with ImPACT (Im-PACT Applications, Inc., Pittsburgh, PA, USA), a computer based test battery consisting of a concussion symptom inventory and six modules measuring neurocognitive function [1], [2]. These modules are used to generate five composite scores (verbal memory, visual memory, visual motor speed, reaction time, and impulse control). The concussion symptom inventory is used to generate a postconcussive symptom score. Good performance is indicated by higher scores, except for reaction time, impulse control, and symptoms where lower scores are better.

### Diffusion tensor imaging

All images were obtained on a Siemens 3T Tim Trio system (Siemens, Malvern, PA, USA). DTI parameters were: TR/TE = 10 s/89 ms, 2×2×2 mm voxel, 60 diffusion directions with b = 1200 s/mm^2^ and one average, b = 0 images with 10 averages. Home-built software based on tools in FSL was used for eddy-current and susceptibility corrections and tensor calculations. For each subject, FA image of each time point was aligned to each other to identify the “most representative” one as the template to achieve spatial co-registration among three longitudinal DTI data. Bootstrap samples were generated to approximate the real situation when numerous repeated measurements were performed. Empirical distributions with 250 bootstrap samples of FA/MD for each longitudinal data of the same subject were generated. Pair-wise statistical comparisons among longitudinal DTI data with permutation t-test (2500 permutations) were conducted based on bootstrap samples using Randomise in FSL. A cluster-based threshold (with a cluster size of 10) was used for multiple-comparison correction. These methods are described elsewhere [28].

### Immunohistochemistry

**Figure 4:**
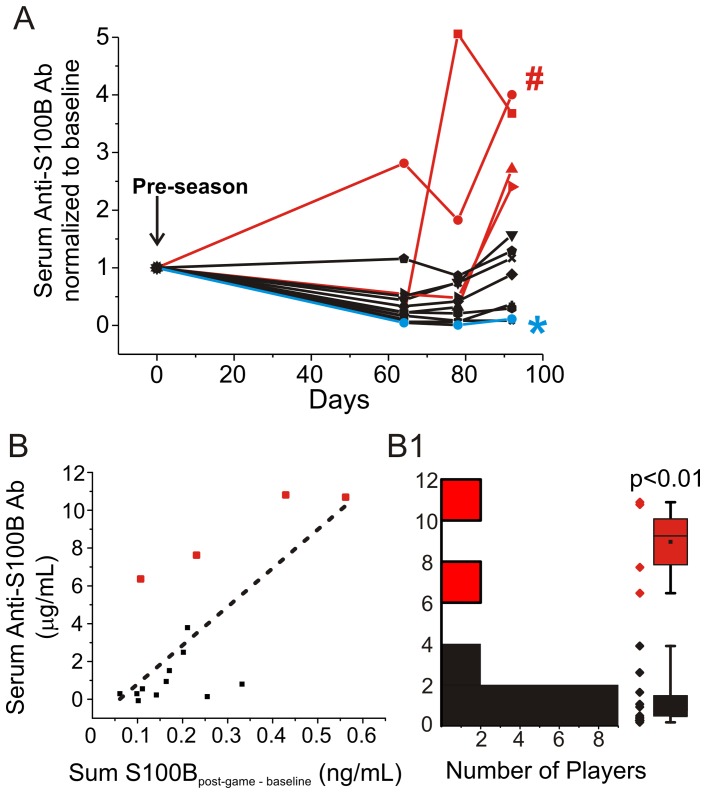
Serum anti-S100B antibody levels in football players before and after a season. **A**) Time course of auto-S100BAb in blood. Auto-S100Ab were measured at baseline and at time (days from the baseline) corresponding to the last three games of the season. Data were normalized by the baseline auto-S100B Ab values. **B**) Significant correlation between serum anti-S100B Ab measured at the end of the season and the cumulative (5 games) S100B_post-game – baseline_ values (see text for details). **C**) Bimodal distribution of players' anti-S100BAb titer at the end of the season. The robustness of the methods employed to detect auto-antibodies was verified as shown in Table S3.

Serum samples from players with the lowest and the highest auto-Ab titers were chosen (see Figure 4). A 50 kDa filtration (14000 RPM for 1 hour at 4°C) was used to separate IgG from low molecular weight proteins. The supernatant and the eluate were collected and protein estimation was performed using the Bradford method. A porcine brain (IACUC Protocol number 08685) was fixed with 4% paraformaldehyde (PFA) followed by 30% sucrose. Using a Leica cryostat, 20–25 μm sections were cut. Immunohistochemistry was performed on porcine brain slices as previously described [29], [30], using human serum samples as “primary” antibody. Serum dilution series was performed (1∶500, 1∶1000 and 1∶5000). Immunostaining was conducted using diaminobenzidine (DAB). Sections were washed in phosphate buffer saline (PBS, 1X), then permeabilized with 0.3% TWEEN/PBS for 1 hour and rinsed. Blocking of endogenous peroxide was done using 0.3% hydrogen peroxide in methanol for 20 minutes. Sections were washed with PBS and then incubated for 1 hour in 5% goat serum followed by overnight incubation with human serum (1∶5000). Sections were then washed with PBS and incubated for 1 hour with a biotinylated anti-human IgG (1∶100. Vector laboratories, Burlingame, CA) prepared in 0.4% Triton-X. Brain sections without human sera were simultaneously processed as a control. Sections were incubated for 1 hour with an avidin/biotin complex (Elite Vectastain ABC Kit; Vector Labs, Burlingame, CA). After PBS washes, the antibody binding sites were visualized using DAB (Peroxidase Substrate Kit, Vector Labs Burlingame, CA) and mounted on slides. The slides were dehydrated and mounted using Permount solution.

### Gel-filtration chromatography – Western blot – Mass spectrometry

Size-exclusion chromatography was performed on human brain homogenate at 4°C. The column was equilibrated at 0.5 ml/min with 40 mM 4-(2-hydroxyethyl)-1-piperazineethanesulfonic acid (EPPS) buffer pH 7.6, containing 3 mM DTT, 10% glycerol, and 150 mM NaCl. A human brain sample (IRB 07–322) processed by fractionating 100 µl of samples (20 µg/µl) on a Superdex 200 gel-filtration column at 4°C. Protein content in the column effluent was detected at 280 nm using a flow-through detector. Western blot analysis was performed to determine the brain targets of serum autoantibodies. Specifically, all fractions (174 kDa to 4 kDa) were loaded on 15% SDS-PAGE gels. Proteins were electroblotted to a PVDF membrane and probed with the specific human serum of the player as primary antibody (dilution 1∶5000, 2 hours at room temperature) and anti-human IgG as secondary antibody (dilution 1∶5000, 1 hour at room temperature). Blots were then rinsed in PBS-Tween then quickly rinsed in dH_2_O to remove phosphate buffer. Blots were then developed using the Pierce enhanced chemiluminescence (ECL) substrate (32106, Pierce, Rockford, IL) and autoradiography film (XAR ALF 1824, Lab Scientific, Livingston, NJ). In a parallel experiment, brain fractions were stained using gel code blue. Selected bands were characterized by Mass Spectrometry. We used a LC-MS system Finnigan LTQ-Obitrap Elite hybrid mass spectrometer system available at the core facility. Five μL volumes of the extract were injected and the peptides eluted from the column by an acetonitrile/0.1% formic acid gradient at a flow rate of 0.25 μL/min. The digest was analyzed using the data dependent multitask capability of the instrument acquiring full scan mass spectra to determine peptide molecular weights and product ion spectra to determine amino acid sequence in successive instrument scans. This mode of analysis produces approximately 15000 collisions and inducing dissociation spectra of ions. Peptide matches were identified using the NCBI database and a human taxonomy filter.

### Statistical Analysis

All data analysis was performed using OriginPro 8.5, JMP Pro 9.0, and SAS. Co-linearity tests of the baseline measures of race, age, BMI, and previous head injury indicated these variables could be used together in a multivariable study to test for correlations with S100B measures. Repeated measures backward elimination regression methods indicted no significant correlations between these baseline measures and the S100B outcomes. A one-way ANOVA was used to compare serum levels pre- and post-game for each contest, as well as the season as a whole. In order to test the effects of hit frequency and severity on serum S100B levels, changes from baseline in post-game S100B were measured against the HHI and the player categories produced by video film review. These tests were run using within-subjects factor ANOVA with Dunnett's correction for comparisons to baseline. All analysis of auto-antibody titer was performed using linear regression t-tests and one-way ANOVA. A p-value <0.05 was considered significant in all cases.

## Results

The demographic characteristics of the subjects enrolled in this study are shown in Figure 1 and Table 1 where the correlation between S100B levels, race, age, body mass index (BMI), history of previous concussion and field positions are reported. Linear regression analysis revealed no correlation between age and S100B levels (Figure 1A; R^2^ = 0.426, P = 0.1236) or BMI and S100B levels (Figure 1B; R^2^ = 0.0217, P = 0.2744). Given the fact that one of the end points of our study was the consequences of sub-concussive episodes, particular care was taken in investigating the history of previous head injury in the players enrolled. The results in Figure 1C show no statistical difference between S100B levels measured in players with a previous history of concussion compared to those who did not report such events (P = 0.61). History of concussion was defined as presence of concussive episodes reported in season before enrollment. Additional data and demographics are in the Table S1. S100B baseline levels did not depend on field position (defensive backs, defensive lineman, offensive lineman, special team, p = 0.5235, see Table 1). The relationship between race and baseline S100B was below our threshold for statistical significance (p = 0.06). This is consistent with findings by others [31].

**Table 1:** Demographics and characteristics of players enrolled for the study.

	University of Rochester (n = 10)	Cleveland Clinic (n = 27)
**Race, No. (%)**		
** Caucasian**	9 (90%)	24 (%89)
** African American**	1 (10%)	3 (%11)
**BMI, mean (SD)**	28.57 (SD 5.6)	31.0 (SD 4.5)
**Previous Head Injury, No. (%)**	1(10%)	18 (67%)
**Position**		
** Offensive Back**	2 (20%)	3 (11)
** Offensive Lineman**	2(20%)	7 (26)
** Defensive Back**	3(30%)	11 (41)
** Defensive Lineman**	3 (30%)	5 (18)
** Special Teams**	0	1 (4)

### Serum S100B changes after football games

Figure 2A shows the changes in serum S100B measured before and after the game. The same data are shown as percent of pre-game values in Figure 2B. In those players where S100B increases were detected, levels returned to pre-game values within 24 hours after the game (Figure S1). Reporting S100B changes as difference between post-game and pre-game produced results that were similar to those obtained when post-game changes were compared with pre-season baseline values. The average S100B_post-game – pre-game_ was 0.045±0.05 ng/ml, while the average S100B_post-game – baseline_ was 0.051±0.05 ng/ml. This further confirms that S100B levels return rapidly to baseline when concussions are not present. There was no statistical difference when comparing S100B_post-game – baseline_ to S100B_post-game – pre-game_ values (P = 0.61). These results also show that blood samples collected prior to a season can be used as reference thus avoiding serial pre-game blood draws. The yellow shaded area in Figure 2A highlights the range of S100B values with a >90% negative predictive value for BBB integrity measured by Gd-MRI [15], the CSF/serum albumin coefficient [14], or mTBI diagnosed by CT (ceilings values are 0.12 or 0.1 ng/ml depending on the test used [32], [33]). The positive predictive value of S100B is poor, since levels above threshold indicate BBBD which is a feature of many neurological diseases [15], [16], [32]. Note that with two exceptions, baseline and pre-game S100B levels were below the ceiling of BBBD. Nine players experienced an above BBBD threshold post-game increase. Only a few players (n = 5) displayed a repetitive (two or more) S100B increase above this threshold.

### S100B changes correlate with number and severity of head hits

Figure 3A shows that players with a HHI of 6 (see Methods for details and Table S2) had a significantly higher S100B_post-game – baseline_ than players with a HHI of 0 (P = 0.03). There was also a significant positive trend (P<0.01) correlating S100B_post-game – baseline_ and HHI. Results obtained from film review (Figure 3B) showed that S100B serum levels increased from baseline only in those players who experienced frequent sub-concussive head hits (SHH, P<0.01) and that just “playing a game” does not affect serum S100B. There was no correlation between serum S100B surges and the number of body contacts/hits.

### Relationship between S100B and anti S100BAb in serum

One of the risk factors associated with repeated head concussions or sub-concussive episodes is cognitive decline [5], [6], [17]. Recent reports by others have shown that in patients affected by senile dementia, one of the early markers of cognitive impairment is the presence of antibodies directed against S100B [21]–[23], [34]. We therefore tested the hypothesis that repetitive increases in serum S100B result in an autoimmune response that can be measured by analyzing the presence of anti-S100BAb in serum. Reproducibility across experiments was confirmed using standard amounts of human anti-S100B antibodies (see Table S3; see Methods). Figure 4A shows the progressive increase in anti-S100Bab titer in serum of approximately 50% of the football players enrolled for the season. Data were normalized by pre-season anti-S100BAb titers. As in the case of S100B serum values, not all players displayed an increase in antibody titers. This was further analyzed by the data plot in Figure 4B. While a significant correlation (R^2^ = 0.54, P<0.01) was found between total S100B serum level increases, this was due to the contribution of a subpopulation of players who had the most extensive S100B increases during the season. The values for S100B during the season are expressed as Σ S100B_post-game – baseline_ measured in 5 games. Figure 4B1 underscores the bimodal distribution of players' anti-S100Ab at the end of the season. Note that the players in 4B and A are coded by red color for emphasis.

### Correlation between auto-antibodies against S100B, radiological indexes and composite tests

**Figure 5:**
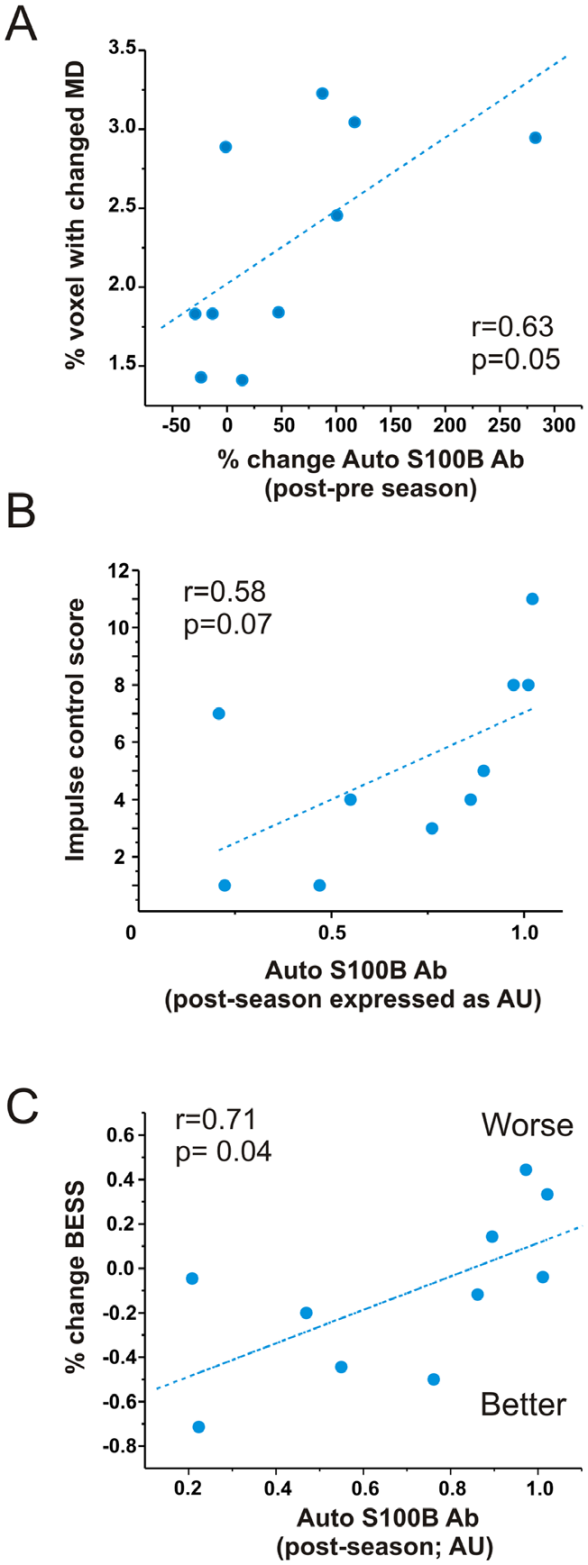
Auto-antibodies in serum correlate with DTI and cognitive changes. **A**) Mean diffusivity index expressed as percent of white matter voxels showing either significant increase or decrease in MD between the preseason and 6 months after the post-season. Titers for antibodies refer to changes between pre- and post-season. In other words, the second antibody measurement was performed six month before the second MRI scan. Note that a correlation exists between these two measures, suggesting a temporal relationship between imaging and auto-immune response. **B**) Absolute values of auto-antibodies (expressed as absorbance) correlate with a worsening impulse control. A comparable relationship was also found between auto-immune titers and decline in postural stability (**C**).

To evaluate possible structural CNS changes, subjects underwent DTI scanning pre- and post-season as well as after 6 months of no head contact. Previous studies [28] have shown that DTI can be used to longitudinally follow post-traumatic changes in football players. We used the same approach and data analysis to test the hypothesis that a correlation exists between serum auto-antibodies for S100B and DTI changes. Of the DTI variables analyzed (see [28]), changes in mean diffusion (MD) correlated most significantly with surges in auto-antibody levels (Figure 5A). We further tested the hypothesis that cognitive changes are also present in those players with highest auto-antibody post-season levels. We found a correlation (p = 0.07, r = 0.58) between impulse control and auto-antibodies (Figure 5B). The impulse control score indicates the total number of errors committed during two specific parts of the test and can result from loss of frontal lobe inhibition that sometimes accompanies concussion. Because postural stablity can also be disturbed after concussion, we used the Balance Error Scoring System (BESS) and compared the results to serology to show a correlation between antibodies in serum and change in balance (Figure 5C).

### Immunohistochemistry detection of serum auto-immunogenicity

**Figure 6:**
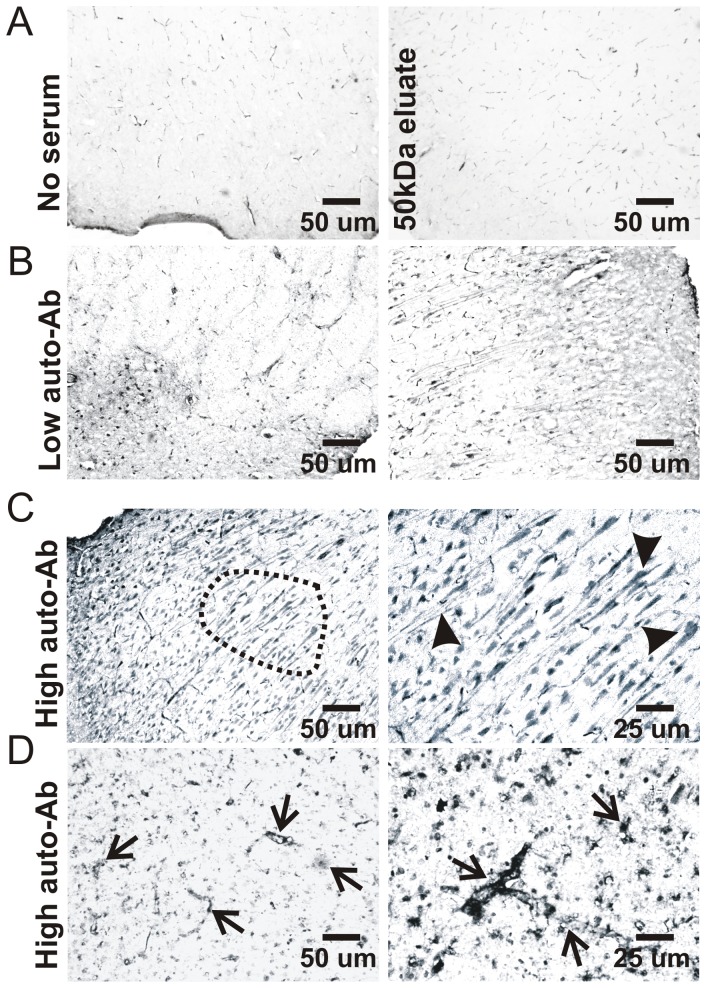
IgG from players with elevated anti-S100B levels recognize glial and neuronal epitopes. Serum samples obtained from selected players (highest and lowest auto-S100B antibody titers, see # and * in Figure 4) where used as “primary” antibody for recognition of brain epitopes. A control porcine brain was used (see text for details). **A**) No signal was observed when serum was omitted or when brain slices were incubated with <50 kDa serum fractions devoid of IgG. **B**) Low levels of brain immune reactivity was observed when using the serum sample from the player with lowest auto-S100B antibody levels. **C–D**) A more pronounced signal was observed when using the serum from the most “auto-immune” sample. Both glial and neuronal staining were stained by these IgGs, supporting the hypothesis that auto-immunity is not limited to the glial protein S100B but rather extends to other neuronal epitopes.

**Figure 7:**
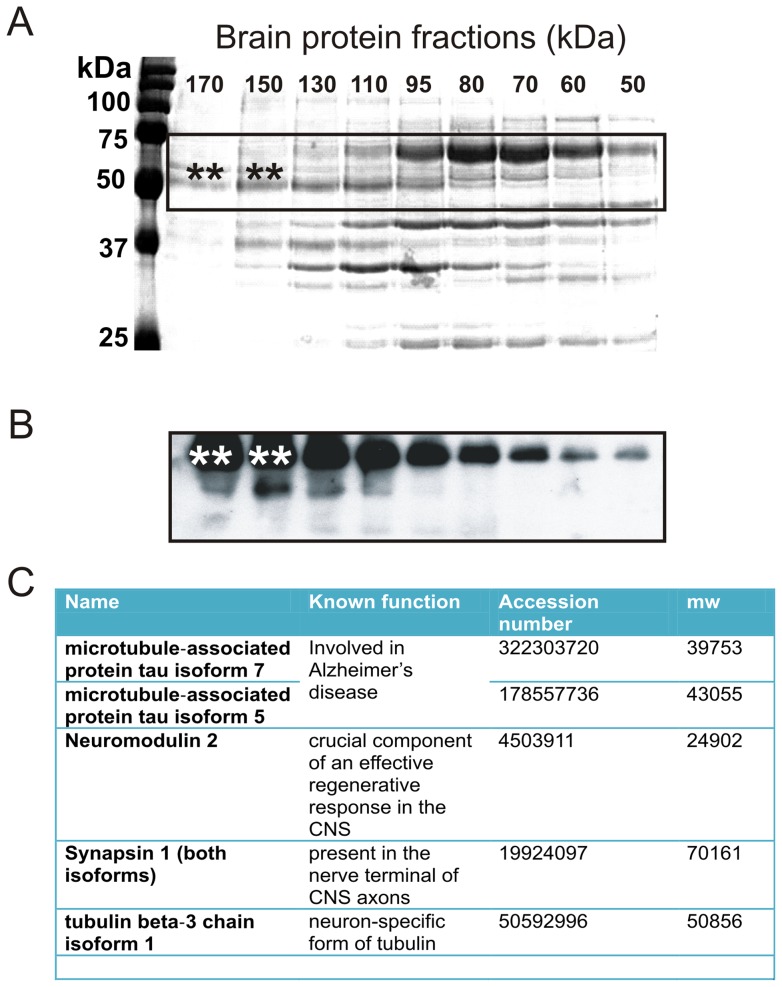
FPLC-Western Blot and Mass spectroscopy screening of auto-Ab candidates. **A**) Control human brain protein content was fractioned by molecular weight using FPLC. Fractions between 170 and 50 kDa are shown on a polyacrilamide gel (15% SDS) to further separate proteins by molecular weight. This two-step technique allows for a comprehensive separation of the total brain protein content. **B**) A parallel gel was run and processed for western blot analysis with player's serum (highest measured auto-S100B antibody, # in Figure 4) used as a “primary” antibody. Bands of interest (*double asterisks*) were excised and processed for MS analysis. Results are shown in the Table (**C**) and described in the text.

We selected sera from 2 players who displayed the highest and the lowest auto-S100BAb titers at the end of the season (see players with # and * in Figure 4A). Serum samples were used as a source of IgG to test against yet unknown epitopes. To achieve this, we used as a target epitope a normal porcine brain. The porcine brain shares anatomical and proteomic similarities with the human brain [35]. No immunocytochemical signal was detected when IgGs were omitted or when serum was filtered at 50 kDa cutoff (Figure 6A). Above background staining was detected with serum from the player with lowest anti-S100B titers (Figure 6B). In contrast, when IgGs from the player with the highest autoimmune titer was used, significant immunoreactivity was observed in perivascular and parenchymal cells (Figures 6D–C). In addition to glial S100B signal, auto-reactivity was found in neurons. We further tested the hypothesis that IgGs are indeed capable of recognizing human brain proteins other than S100B. The same brain protein source was fractioned by FPLC (50–170 kDa). Each fraction (Figure 7A) was then probed on a gel with IgGs from the most autoimmune serum (Figure 7B). We detected bands in the 50–75 kDa range which when excised from the gel and processed by mass-spectroscopy revealed an array of possible epitopes (see table in Figure 7.

## Discussion

In this study we have shown that elevations of serum S100B indicating blood-brain barrier disruption occur in football players who experience sub-concussive head hits (SHH) below the threshold for a diagnosis of concussion. We have also shown that players who during a season experience the most significant repeated elevations of S100B are also those with the highest titer of serum S100B auto-antibodies. In addition, we were able to correlate these findings to functional and imaging data, both demonstrating that serum auto-antibodies may be related to DTI and cognitive changes. Our data interpretation hinges on the notion that the elevations of S100B we measured in players with the most SHH indicate blood-brain barrier disruption. With the test used for this study, the ceiling for changes indicative of BBBD is 0.12 ng/ml (shaded area in Figure 2). The extent of these changes has been shown to have 0.875 specificity for BBBD measured by albumin coefficient [14] and >0.90 sensitivity for enhancement on MRI [15], [16].

A single concussion can result in a variety of adverse sequelae such as post-concussive symptoms and cognitive dysfunction. Multiple concussions have been linked to chronic traumatic encephalopathy [24], [26], [36]. Although axonal injury is thought to be the anatomical abnormality that underlies these problems [37], there is currently no way to detect this in vivo. We therefore also lack the tools to treat this condition. Diffusion tensor imaging (DTI) can potentially be used to diagnose axonal injury acutely. After mild TBI, fractional anisotropy (FA) decreases and mean diffusion (MD) increases [38]. Since serum markers and DTI have been used to diagnose or detect mTBI, it is surprising that no correlation between these two independent measures has been studied. One of the goals of our study was to compare results obtained with serum markers of blood-brain barrier disruption and their autoantibody counterpart to DTI changes following mTBI or SHH.

### Serum levels of S100B are increased after a game

The clinical significance of elevation of S100B, and therefore of BBBD, measured in players who are not diagnosed with a concussion remains unclear. Our findings show that elevations in S100B correlated with the occurrence of head hits and their intensity (Figure 3). This suggests that BBBD follows repeated non-concussive episodes during a game. Thus, S100B may be useful to objectively quantify risk for subsequent pathological sequelae. We are aware that the questionnaire used (see Methods and Table S2) and the film reviewing processes were not devoid from pitfall. We recognize that standardize methods are available to assess player conditions, including SCAT2 and balance tests. However, time and physical constrains are to be taken into account during the post-game serum sapling; the test we used was designed to fit such a time frame and the locker room settings. A parallel effort by the team at the University of Rochester reported that a correlation exists between objective, telemetry-based indices of HHI and serum S100B levels (*data not shown*).

The possibility that increased serum levels of S100B was due to events unrelated to SHH is not supported by our findings but has been previously suggested [39]–[41]. Our data show that players who did not experience significant head hits or players who remained at the sidelines had S100B levels significantly lower than those measured in players who experienced head hits (Figure 3). We also show that S100B increases in serum cannot be ascribed to soft tissue injury or other aspects of a football game unrelated to head hits. The lack of influence of “just playing a game” on S100B serum levels is perhaps surprising given that S100B is found in extra-cranial tissues (please see [39], [40]).

### Auto-antibodies levels correlate with serum S100B and blood-brain barrier disruption

Our data show that repetitive increases of serum S100B levels over a season are sufficient to trigger the production of anti-S100BAb. We found the highest titers of anti-S100BAb in players with the highest S100B_post-game – baseline_ throughout the season (Figure 4B). In a parallel study, we showed that S100B accumulates in dendritic cells, the chief regulators of auto-immune responses (*not shown*). While S100B protein is normally segregated in the brain, its increase in blood may be perceived as non-self antigen by dendritic cells. This finding could provide a biological rationale for the auto-immune response to S100B. In addition, we report that human S100B protein has unique auto-antigenic properties since it contains a sequence of 10 aminoacids (sequence: *elinnelshf*) that is not present in any other human protein. This sequence is in a crucial position accessible to detection by dendritic cells even when S100B is bound to other protein. However, our results suggest that, beside antibodies for S100B, other auto-antibodies may be present in blood of players. In fact, we detected IgG which target both glial and neuronal self-epitopes. Mass spectroscopy revealed putative brain targets such as synapsin 1, microtubule-associated proteins for Tau isoforms, and the specific brain tubulin beta-3 (see Figure 7). These proteins are involved in neuronal physiology and transmission and have been proposed to participate to the progression of neurological disorders [30]. These data call for an in depth analysis of the serum auto-immune load against brain proteins in subjects that have experienced significant BBBD.

### Clinical significance of auto-antibodies against S100B

A link between cognitive decline and American football has been extensively studied and confirmed [24]. However, the gap between a player's career and the earliest onset of signs of cognitive changes spans many decades making a direct link between the two difficult to study. It is possible that repeated “opening” of the BBB result in a delayed pathological response consisting of immune cell activation by a brain antigen perceived as foreign (see also consideration on S100B structure above). While our results are limited to the astrocytic protein S100B a similar response may be directed towards other antigens present in the brain, including myelin basic protein, tau, beta-trace protein, *etc*.

The presence of CNS auto-antibodies does not necessarily translate into an autoimmune or neurological disease. In fact, auto-antibodies can be detected in individuals who do not have any autoimmune symptoms [22]. Auto-antibodies may become pathogenic upon penetration into the CNS *via* either a “leaky” BBB or extravasation into CSF from the sub-arachnoid space [10]. Elevated antibody titers for S100B and other brain protein have been described in a variety of human diseases including Alzheimer's dementia [21], [34], [42]. We hypothesize that the presence of an autoimmune response that lingers over time following repeated BBBD during adulthood may cause an early cognitive decline. This response may be characterized by influx of immunoglobulins into the brain or under the most pathogenic conditions lymphocyte entry into CNS. Presence of immunoglobulin in brain has been demonstrated in several CNS disorders, including epilepsy [43].

We hypothesize that a number of molecular players “switch teams” when the BBB is breached [44]. Molecular players in serum (albumin, magnesium ions, potassium ions, immunoglobulins) may enter the brain after BBBD and create havoc in CNS homeostasis. Conversely, brain protein may trigger, as shown here, an autoimmune response by antigen unmasking. If the cause of auto-IgG is multiple head hits and S100B leakage into serum, it is not surprising that football players can develop auto-antibody against S100B after a single season. In fact, a typical player experiences upward of 1,000 head hits (and perhaps BBBD) each season [45]. Others have suggested that even subconcussive events, when repeated, are potentially pathogenic [46]. However, not all results support this hypothesis, at least in football [47]. Our data show that in virtual absence of clinical signs or symptoms, MR-DTI and BESS changes consistent with mild functional impairment can be measured in a subpopulation of players. Relevant to the etiology of these changes is the predictive value of: 1) Markers of blood-brain barrier disruption such as S100B appearing in blood after each game (Figure 2); 2) Number of subconcussive head hits (Figure 3); 3) Delayed (Figure 4) onset of auto-immune titers. Thus, DTI and BESS results correlated significantly with auto-antibodies blood levels which in turn depend on head hits and BBBD. Our results thus support the notion that even in the absence of a frank concussion, repetitive sub-concussive head hits can lead to subtle pathological changes detectable by imaging and behavioral testing.

In conclusion, we have shown that serum S100B elevations, which are indicators of BBBD, occur in football players even when concussions are not diagnosed. Our results suggest that these levels of S100B trigger production of auto-antibodies that may constitute a risk factor for premature neurodegeneration.

## Supporting Information

Figure S1 **Transient nature of S100B increases after a football game.** Data refer to one game and are plotted as absolute S100B serum values (ng/ml, **A1**) or normalized by pre-game levels (**A2**). Note that in the absence of concussion, S100B serum levels return to pre-game baseline 24 hours after the game. While the overall differences between pre-, post- and 24 hours post-game were not statistically significant (**A1**), 5 players had an increased in S100B of 2 times (200%) their respective baselines (**A2**).
(TIF)

Figure S2 **Validation of the auto-immune tests used to detect S100B autoantibodies.** See also Methods.
(TIF)

Table S1 **Demographic characteristics of the players enrolled for the results in Figure S1.**
(DOC)

Table S2 **Parameters of Head Hit Index (HHI) calculations.** A score system was used to segregate players based on the number and intensity of head hits experienced during games (see also Methods for details).
(DOC)

Table S3 **Reproducibility of reverse ELISA experiments used to measure antibodies against S100B in serum of athletes (n = 5 repeats).** Commercially available human S100B was used to coat the wells; the standard used to transform absorbance in µg/ml consisted of human monoclonal anti-S100B antibodies. The mean absorbance and its variability (Standard Deviation values and its %) are shown. Data were obtained by calibrating the system with human monoclonal anti-S100B antibodies. ELISA wells were coated with human S100B, 1 µg/well.
(DOC)
