# Supplementary figures and images for: Consequences of Repeated Blood-Brain Barrier Disruption in Football Players
Source: PLoS One. 2013 Mar 6;8(3):e56805. doi: 10.1371/journal.pone.0056805 (PMC3590196; doi:10.1371/journal.pone.0056805)

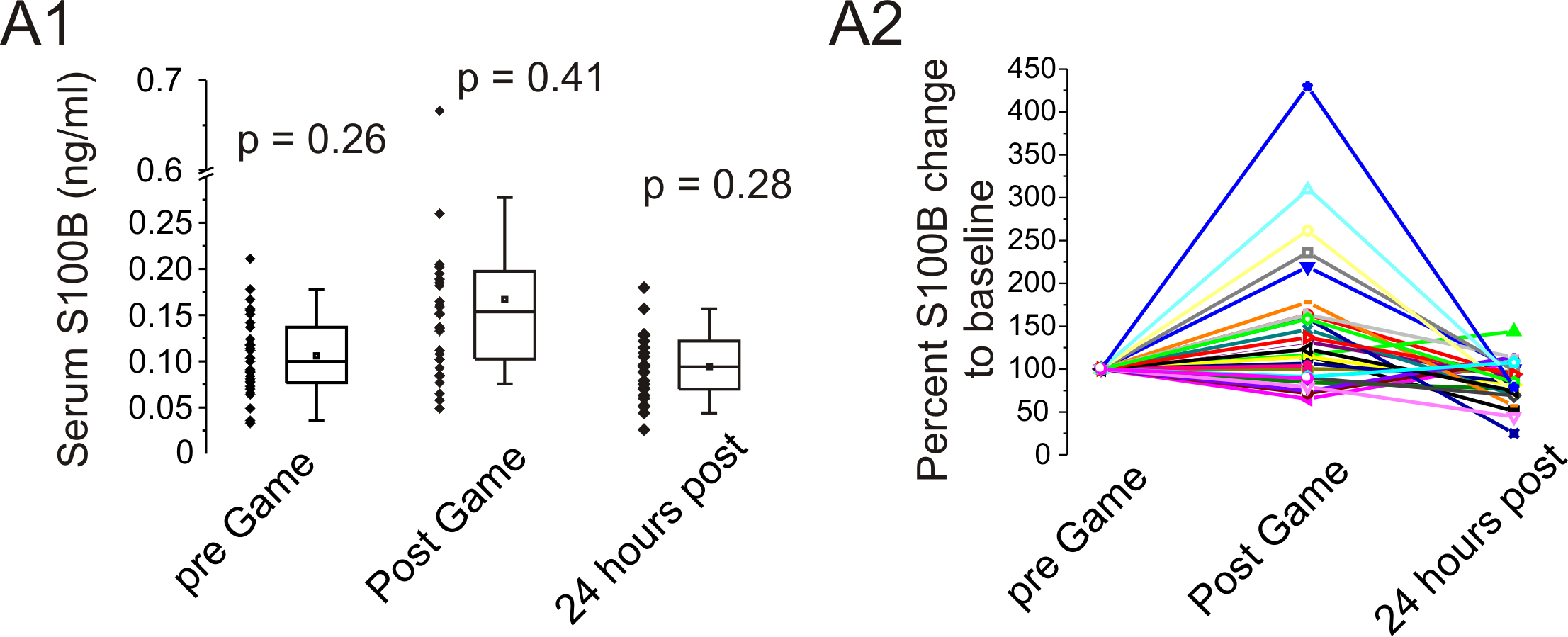

Supplement: Figure S1 — Transient nature of S100B increases after a football game. Data refer to one game and are plotted as absolute S100B serum values (ng/ml, A1) or normalized by pre-game levels (A2). Note that in the absence of concussion, S100B serum levels return to pre-game baseline 24 hours after the game. While the overall differences between pre-, post- and 24 hours post-game were not statistically significant (A1), 5 players had an increased in S100B of 2 times (200%) their respective baselines (A2). (TIF) [file pone.0056805.s001.tif]

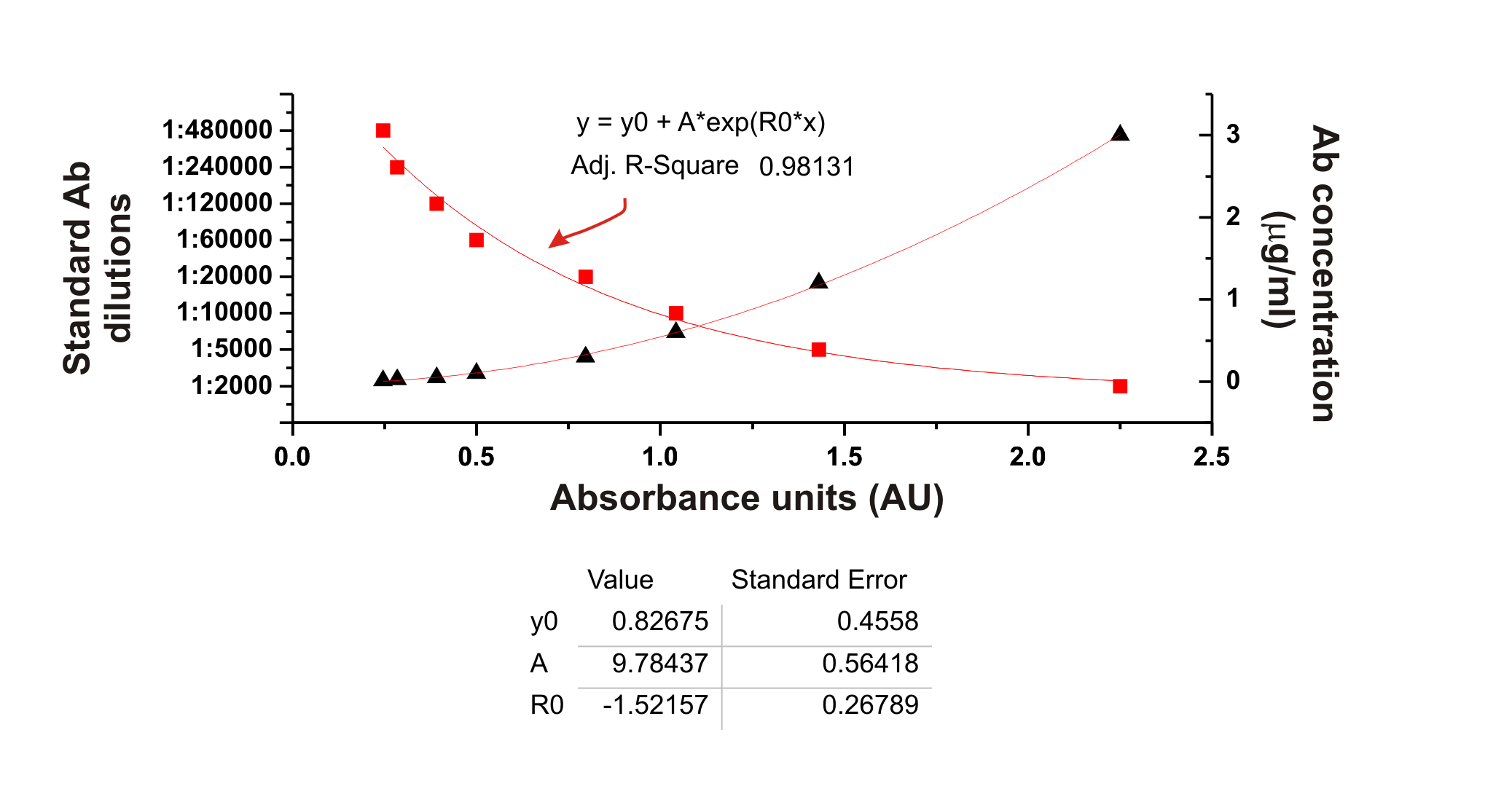

Supplement: Figure S2 — Validation of the auto-immune tests used to detect S100B autoantibodies. See also Methods. (TIF) [file pone.0056805.s002.tif]
