# Supplementary material for: Consequences of Repeated Blood-Brain Barrier Disruption in Football Players
Source: PLoS One. 2013 Mar 6;8(3):e56805. doi: 10.1371/journal.pone.0056805 (PMC3590196; doi:10.1371/journal.pone.0056805)
Supplement: Table S1 — Demographic characteristics of the players enrolled for the results in Figure S1. (DOC) [file pone.0056805.s003.doc]

|  | **Data relevant to supplemental Fig. 1 (n=30)** |
| --- | --- |
| Age, mean (SD), yr | 20.8 (1.3) |
| Race, No. (%) |  |
| Caucasian | 24 (80) |
| African American | 6 (20) |
| BMI, mean (SD) | 28.1 (4.5) |
| Previous Head Injury, No. (%) | 10 (33) |
| Position |  |
| Offensive Back | 9 (31) |
| Offensive Lineman | 4 (14) |
| Defensive Back | 9 (31) |
| Defensive Lineman | 6 (21) |
| Special Teams | 1 (3) |

**Table S1:** Demographic characteristics of the players enrolled for the results in Supplemental Figure 1.
