# Supplementary material for: Consequences of Repeated Blood-Brain Barrier Disruption in Football Players
Source: PLoS One. 2013 Mar 6;8(3):e56805. doi: 10.1371/journal.pone.0056805 (PMC3590196; doi:10.1371/journal.pone.0056805)
Supplement: Table S2 — Parameters of Head Hit Index (HHI) calculations. A score system was used to segregate players based on the number and intensity of head hits experienced during games (see also Methods for details). (DOC) [file pone.0056805.s004.doc]

| **HHI**  **(A x B)** | **A = 0**  (no hits) | **A = 1**  (1 - 4 hits) | **A = 2**  (5 - 20 hits) | **A = 3**  (>20 hits) |
| --- | --- | --- | --- | --- |
| **B = 0**  (e.g., negligible, body-helmet or ground-helmet contacts ) | 0 - no head hits | 0- negligible hits | 0 - negligible hits | N.A. |
| **B = 1**  (e.g., player acknowledges the head hit) | 0 - no head hits | 1 - e.g., a few head hits during game | 2 - several but normal hits | 3 - players who had > 20 normal hits |
| **B = 2**  (players distinctively remember the head hit (e.g., helmet-to-helmet) | 0 - no head hits | 2- e.g., few harsh hits | 4 - player who had several hits including harsh ones | 6 - players who had > 20 hits of which several were harsh |

**Table S2:** Parameters of Head Hit Index (HHI) calculations. A score system was used to segregate players based on the number and intensity of head hits experienced during games (see also Methods for details).
