# Supplementary material for: Consequences of Repeated Blood-Brain Barrier Disruption in Football Players
Source: PLoS One. 2013 Mar 6;8(3):e56805. doi: 10.1371/journal.pone.0056805 (PMC3590196; doi:10.1371/journal.pone.0056805)
Supplement: Table S3 — Reproducibility of reverse ELISA experiments used to measure antibodies against S100B in serum of athletes (n = 5 repeats). Commercially available human S100B was used to coat the wells; the standard used to transform absorbance in µg/ml consisted of human monoclonal anti-S100B antibodies. The mean absorbance and its variability (Standard Deviation values and its %) are shown. Data were obtained by calibrating the system with human monoclonal anti-S100B antibodies. ELISA wells were coated with human S100B, 1 µg/well. (DOC) [file pone.0056805.s005.doc]

**Table S3:** Reproducibility of reverse ELISA experiments used to measure antibodies against S100B in serum of athletes (n = 5 repeats). Commercially available human S100B was used to coat the wells; the standard used to transform absorbance in µg/ml consisted of human monoclonal anti-S100B antibodies. The mean absorbance and its variability (Standard Deviation values and its %) are shown. Data were obtained by calibrating the system with human monoclonal anti-S100B antibodies. ELISA wells were coated with human S100B, 1µg/well.

| **Anti-S100B calibrating antibody (μg)** | **Anti-S100B STD (dilutions)** | **Mean Absorbance** | **SD** | **% SD / Absorbance** |
| --- | --- | --- | --- | --- |
| 3 | 1:2000 | 2.25 | 0.03962 | 1.76092 |
| 1.2 | 1:5000 | 1.431 | 0.21744 | 15.19515 |
| 0.6 | 1:10000 | 1.0422 | 0.18231 | 17.49317 |
| 0.3 | 1:20000 | 0.7964 | 0.20163 | 25.31706 |
| 0.1 | 1:60000 | 0.4996 | 0.11358 | 22.7344 |
| 0.05 | 1:120000 | 0.3916 | 0.04821 | 12.31091 |
| 0.025 | 1:240000 | 0.2844 | 0.04184 | 14.71073 |
| 0.0125 | 1:480000 | 0.2462 | 0.04691 | 19.05185 |
